# Acute Pulmonary Embolism Severity Assessment Evaluated with Dual Energy CT Perfusion Compared to Conventional CT Angiographic Measurements

**DOI:** 10.3390/diagnostics11030495

**Published:** 2021-03-11

**Authors:** Samir Jawad, Peter Sommer Ulriksen, Anna Kalhauge, Kristoffer Lindskov Hansen

**Affiliations:** 1 Department of Radiology, Copenhagen University Hospital, Rigshospitalet, 2100 Copenhagen, Denmark; peter.sommer.ulriksen@regionh.dk (P.S.U.); anna.kalhauge@regionh.dk (A.K.); kristoffer.lindskov.hansen.01@regionh.dk (K.L.H.); 2 Department of Clinical Medicine, University of Copenhagen, 2100 Copenhagen, Denmark

**Keywords:** pulmonary embolism, Dual Energy CT, lung perfusion, pulmonary perfusion, Dual Source CT

## Abstract

The purpose of the study was to investigate whether Dual Energy CT (DECT) can be used as a diagnostic tool to assess the severity of acute pulmonary embolism (PE) by correlating parenchymal perfusion defect volume, obstruction score and right ventricular-to-left ventricular (RV/LV) diameter ratio using CT angiography (CTA) and DECT perfusion imaging. A total of 43 patients who underwent CTA and DECT perfusion imaging with clinical suspicion of acute PE were retrospectively included in the study. In total, 25 of these patients had acute PE findings on CTA. DECT assessed perfusion defect volume (PDvol) were automatically and semiautomatically quantified. Overall, two CTA methods for risk assessment in patients with acute PE were assessed: the RV/LV diameter ratio and the Modified Miller obstruction score. Automatic PDvol had a weak correlation (*r* = 0.47, *p* = 0.02) and semiautomatic PDvol (*r* = 0.68, *p* < 0.001) had a moderate correlation to obstruction score in patients with confirmed acute PE, while only semiautomatic PDvol (*r* = 0.43, *p* = 0.03) had a weak correlation with the RV/LV diameter ratio. Our data indicate that PDvol assessed by DECT software technique may be a helpful tool to assess the severity of acute PE when compared to obstruction score and RV/LV diameter ratio.

## 1. Introduction

Pulmonary embolism (PE) is a common clinical condition being third on the list of cardiovascular causes of death [1]. Severe PE may lead to hemodynamic instability with right ventricle dysfunction (RVD) and eventually death. In the acute setting of PE, a prompt diagnosis and risk stratification are of utter importance, as high-risk patients may benefit from intensive care treatment, trombectomy, or thrombolysis [2,3].

Computed tomography angiography (CTA) is considered the first-line imaging modality for suspected acute PE [4]. CTA can also be used to identify a possible differential diagnosis and for risk stratification when PE is diagnosed. Two methods for risk assessment in patients with acute PE with CTA are right ventricular-to-left ventricular (RV/LV) diameter ratio as an indicator of RVD and obstruction score as an indicator of clot burden [3]. The RV/LV diameter ratio has been shown to correlate well with both PE severity and mortality [5], while there is conflicting evidence regarding correlation of CTA obstruction score with RVD and outcome severity [6,7]. Because of this uncertainty, interpersonal variation and increased reading times, CTA obstruction score is rarely used in clinical practice [2].

Dual-energy computed tomography (DECT) has made it possible to simultaneously obtain angiographic CT imaging and pulmonary perfusion maps due to the ability to visualize contrast (iodine) distribution within the pulmonary parenchyma. A number of studies have demonstrated the potential of DECT in the diagnosis of acute PE by manual visualization of wedge-shaped pulmonary perfusion defects [8,9,10].

Similar to CTA obstruction score, there have been developed scoring systems for perfusion defects using manual measurement on DECT iodine distribution maps [11,12]. DECT software rendering can automatically estimate total lung volume (TLV) and perfusion defect volume (PDvol), and hereby reduce the reading time and inter- and intra-observer variation.

The aim of this study was to investigate whether PDvol measured by an automatic and semiautomatic DECT software technique can be used as a diagnostic tool to assess the severity of acute PE by correlating PDvol with CTA obstruction score and RV/LV diameter ratio.

## 2. Materials and Methods

### 2.1. Patients

The study is a retrospective single-center study. The institutional review board at our institution approved the study, and informed patient consent was waived due to the retrospective nature of this study (Ref. No. 20036558).

**Figure 1:**
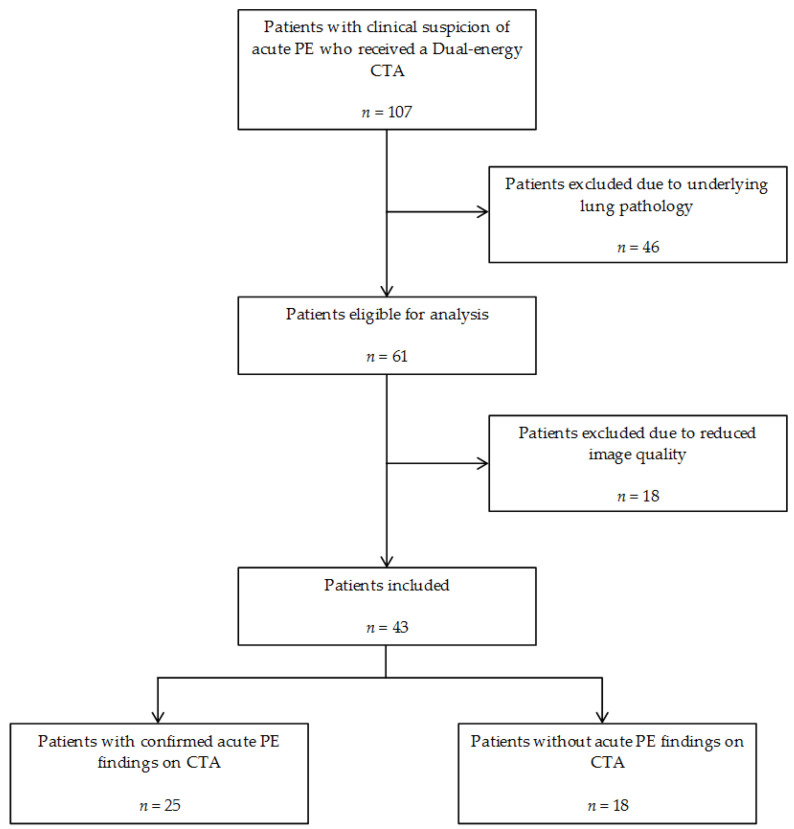
Flowchart of patient inclusion and exclusion. CTA = CT angiography, PE = Pulmonary embolism.

Between August 2019 and January 2021, 107 patients with the clinical suspicion of acute PE received a Dual-energy CTA at our department. A total of 64 were excluded, 46 patients due to underlying lung pathology and 18 patients due to reduced image quality of the DECT or CTA scan that potentially could affect the evaluation of pulmonary perfusion, obstruction score, or RV/LV diameter ratio, e.g., artifacts, insufficient vascular enhancement, metastatic pulmonary neoplasm, pulmonary infection, and pleural effusion. Overall, 43 patients (18 females, mean age 66.5 years, range 30 to 86 years) were finally included. In total, 25 of the included patients were confirmed with acute PE findings on CTA, while 18 patients did not have any CTA findings associated with acute PE (Figure 1).

### 2.2. CT Acquisition

All CT scans were performed on a single source DECT scanner (GE Healthcare Revolution CT, Milwaukee, WI, USA). Tube voltage switched between 80 and 140 kV, and images were acquired in a single breath hold in the caudo-cranial direction from under the diaphragm to the lung apex. A 62 keV virtual monoenergetic dataset for pulmonary CTA series was simulated. A split bolus was injected into an antecubital vein through a power injector: first bolus consist of 35 mL Omnipaque 350mgI/mL, (GE Healthcare, Milwaukee, WI, USA) followed by 15 mL saline solution and second bolus of 15 mL Omnipaque 350 mgI/mL, followed by 30 mL saline solution. Injection flow rate was 5.0 mL/s. A bolus-tracking technique was applied; imaging was automatically initiated 4.6 s after the enhancement level reached the threshold of 120 Hounsfield Units in the region of interest (placed in the pulmonary trunk). Detector collimation was set to 80 mm, gantry rotation time was 0.5 s, pitch value was 1.531, scan field of view was 500 mm, display field of view depended on the patient size, and reconstruction with a slice thickness of 0.625 mm, and an increment of 0.625 mm.

### 2.3. CTA Interpretation

One fifth-year radiology resident (SJ) evaluated all CTA studies on a PACS workstation (AGFA Healthcare, Impax, v6.7.0.6011, Mortsel, Belgium) without any awareness of the initial CTA results and without the knowledge of iodine perfusion maps, PDvol or RV/LV diameter ratio. The presence of filling defects in pulmonary arteries on CTA in patients with relevant clinical suspicion was used to diagnose acute PE. The presence of filling defects in the pulmonary arteries, degree of obstruction and their locations were recorded. The same radiologist reviewed RV/LV diameter ratio 2 weeks after assessing the obstruction scores with the scans in random order.

### 2.4. CTA Obstruction Score

**Figure 2:**
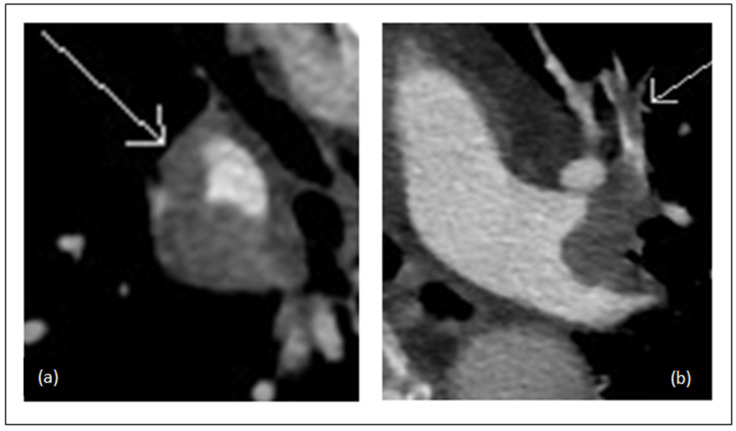
CT angiography in a patient with acute pulmonary embolism. (**a**) Partial occluding thrombus in right basal trunk (Modified Miller score = 4); (**b**) Partial occluding thrombus in the left apical anterior segmental artery (Modified Miller score = 1).

The CTA obstruction score was calculated using the modified Miller (MM) score, as described by Qanadli et al. [13]. This scoring system considers each lung as having 10 segmental arteries (three to the upper lobe, two to the middle lobe and lingula, and five to the lower lobe). The degree of obstruction was assessed as stated: 0 for no obstruction; 1 for partial obstruction; and 2 for complete obstruction (Figure 2). This scoring system differentiates between obstruction in segmental arteries and in seven major arteries that are given a weighting factor depending on the amount of distally arising segmental arteries. Total score range from 0 to 40.

### 2.5. RVD Assessment

**Figure 3:**
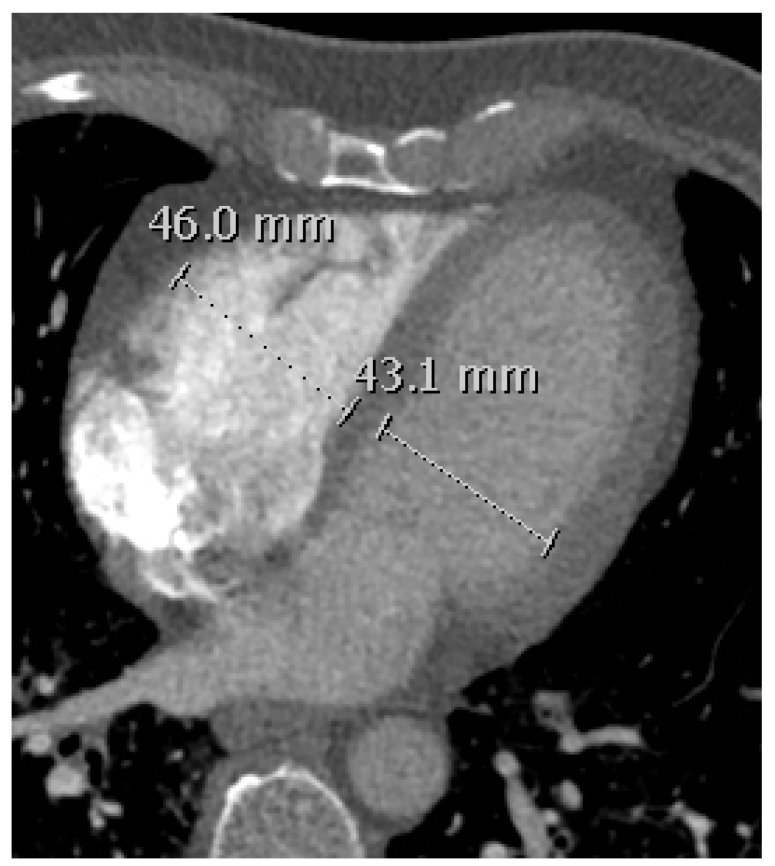
Measurement of right and left ventricle diameter using the axial method on a transverse CT angiography.

RV/LV diameter ratio was assessed using the axial (transverse) method [14]. Both right and left ventricle diameter were measured perpendicular to the long axis of the heart from the interventricular septum to the free outer wall (Figure 3). Previous studies have shown that axial view for assessing RV/LV diameter ratio is comparable to the more time-consuming four chamber reformatted RV/LV diameter ratio in patients with acute PE [15,16,17] or for predicting mortality in PE patients [17].

### 2.6. Quantification of Total Lung Volume and PDvol

Automated quantification of TLV and PDvol were assessed using the commercially available image post-processing Dual Energy software (GE Healthcare, Thoracic VCAR, GSI pulmonary perfusion, AW Server, v3.2 Ext 2.0, Milwaukee, WI, USA) and a GE Healthcare AW workstation (Milwaukee, WI, USA).

**Figure 4:**
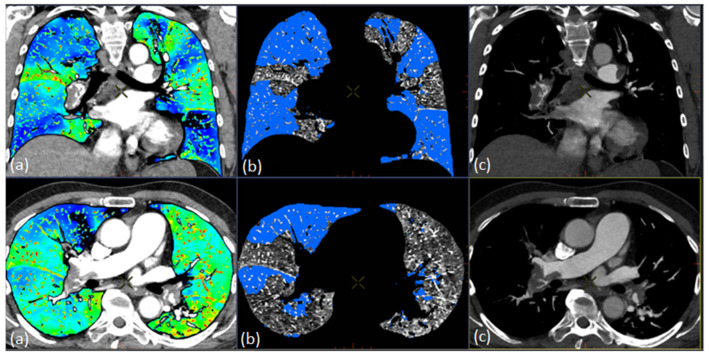
Dual-energy CT, Thoracic VCAR application, gemstone spectral imaging (GSI) pulmonary perfusion in a patient with acute pulmonary embolism. (**a**) Visualization of pulmonary perfusion by colorized iodine overlay. (**b**) Automatic segmentation showing regions (blue areas) representing relative perfusion deficit. (**c**) Monochromatic input volume.

The thoracic VCAR application with GSI pulmonary perfusion can be used to visualize pulmonary perfusion by showing colorized iodine overlay of the lung parenchyma and to identify regions with relative perfusion deficit based on the iodine concentration in that region (Figure 4). The software calculates an automatic threshold value based on an algorithm to represent lung volume with the lowest iodine concentration in the patient. Areas in the lung parenchyma where iodine concentration is lower than the stated threshold value are presented as relative perfusion deficit (perfusion defect) [18]. This threshold can be manually adjusted, though not adjusted for when assessing automatic pulmonary perfusion. The automatic pulmonary perfusion allows for a one-click analysis of estimated TLV and PDvol. While TLV is quantified in cm^3^, PDvol is given both in cm^3^ and as a ratio in percentage when normalized to TLV (PDvol cm^3^/TLV cm^3^). In this study, PDvol normalized to TLV is used for the analyses.

Furthermore, a semiautomatic approach for quantification of TLV and PDvol was assessed, where the only difference from the automatic quantification method was a manual adjustment of the threshold value. One radiologist (PSU), who had 11 years of clinical experience, blindly adjusted the threshold value based on visual evaluation on the colorized iodine overlay maps (Figure 4) to either include hypo-perfused wedge-shaped areas as relative perfusion deficit or to exclude random artefactual non wedge-shaped areas.

### 2.7. Statistical Analysis

Statistical analysis was performed with the use of IBM SPSS Statistics v25 (SPSS Inc., Chicago, IL, USA). Pearson correlation coefficient (*r*) was used to assess agreement between PDvol, CTA obstruction score and RV/LV diameter ratio. Correlation coefficient (*r*) values were interpreted as follows: < 0.20 (no association), 0.20–0.49 (weak), 0.50–0.79 (moderate), and > 0.80 (strong) [19]. Independent sample *t*-test was used to evaluate the difference between the group with confirmed acute PE findings, and the group without acute PE findings. The two tailed *p*-value < 0.05 was considered statistically significant.

## 3. Results

### 3.1. Correlation Between PDvol, RV/LV Diameter and CTA Obstruction Score in all Patients (n = 43)

**Figure 5:**
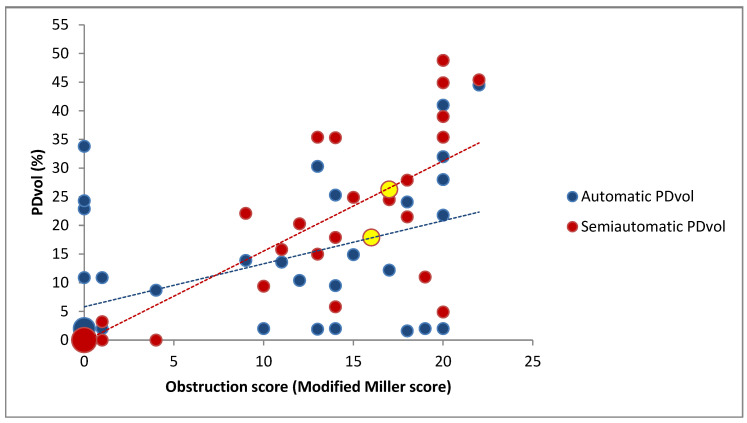
Scatterplot of obstruction score and perfusion defect volume (PDvol) (*n* = 43). Line of best fit is shown with a dotted line. The large red dot in the left corner of the scatterplot represents 18 patients, while the large blue dot in the left corner represents 14 patients. The yellow dots represent patients with identical automatic and semiautomatic PDvol.

**Figure 6:**
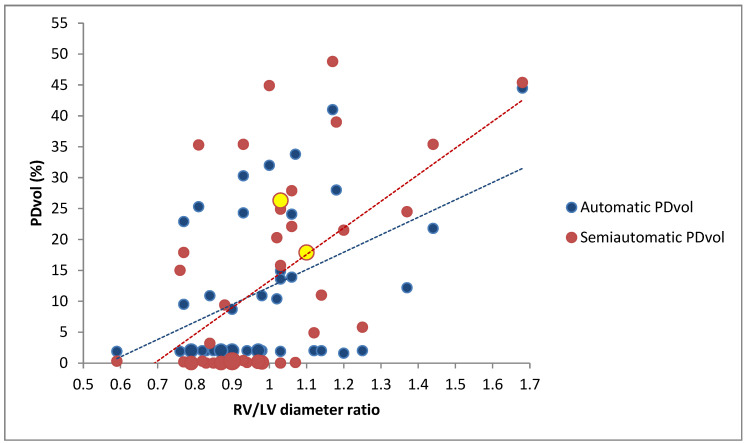
Scatterplot of right ventricular-to-left ventricular (RV/LV) diameter ratio and perfusion defect volume (PDvol) (*n* = 43). Line of best fit is shown with a dotted line. The slightly larger blue and red dots represent 2 or 3 patients, depending on the dot size. The yellow dots represent patients with identical automatic and semiautomatic PDvol.

Automatic PDvol had a moderate correlation with MM score (*r* = 0.51, *p* < 0.001) (Figure 5), and a weak correlation with RV/LV diameter ratio (*r* = 0.45, *p* = 0.002) (Figure 6). Semiautomatic PDvol had a strong correlation with MM score (*r* = 0.85, *p* < 0.001) (Figure 5), and a moderate correlation with RV/LV diameter ratio (*r* = 0.56, *p* < 0.001) (Figure 6). RV/LV diameter ratio had a moderate correlation with MM score (*r* = 0.64, *p* < 0.001).

### 3.2. Correlation Between PDvol, RV/LV Diameter and CTA Obstruction Score in Patients with Confirmed Acute PE (n = 25)

Automatic PDvol had a weak correlation with MM score (*r* = 0.47, *p* = 0.02), but a non-significant correlation with RV/LV diameter ratio (*r* = 0.39, *p* = 0.06). Semiautomatic PDvol had a moderate correlation with MM score (*r* = 0.68, *p* < 0.001), and a weak correlation with RV/LV diameter ratio (*r* = 0.43, *p* = 0.03), while RV/LV diameter ratio had a moderate correlation with MM score (*r* = 0.64, *p* = 0.001).

### 3.3. Difference Between the Group with Confirmed Acute PE (n = 25) and the Group without Acute PE (n = 18)

Automatic PDvol, semiautomatic PDvol and RV/LV diameter ratio were significantly higher in the acute PE group compared with the group without acute PE findings using MM score as the reference (Automatic PDvol *p* < 0.01; semiautomatic PDvol *p* < 0.001; RV/LV diameter ratio *p* < 0.002).

## 4. Discussion

Our results indicate that PDvol assessed by automatic and semiautomatic DECT software technique may be a helpful tool to assess the severity of acute PE. In patients with confirmed acute PE, automatic quantified PDvol had a weak correlation and semiautomatic quantified PDvol had a moderate correlation to obstruction score, while only semiautomatic quantified PDvol was correlated with the main CTA marker for PE severity and death, i.e., RV/LV diameter ratio though with a weak association [5]. When evaluating all patients suspected for acute PE, automatic and semiautomatic quantified PDvol had a moderate and a strong correlation to obstruction score and a weak and a moderate correlation to RV/LV ratio, respectively. PDvol and RV/LV diameter ratio were significantly different in patients with acute PE compared to patients without acute PE.

Apfaltrer et al. and Meinel et al., also used automatic or semiautomatic quantification of pulmonary perfusion to assess acute PE severity by correlating pulmonary perfusion with obstruction score and RV/LV diameter ratio [2,20]. With similar results to ours, Meinel et al. showed that pulmonary perfused blood volume can be used to stratify the risk for ICU admission in acute PE, as automatically quantified pulmonary perfused blood volume showed a moderate negative correlation (*r* = −0.46) with obstruction score (MM score), axial RV/LV diameter ratio (*r* = −0.52) and serum troponin I (*r* = −0.45) [2]. In contrast, Apfaltrer et al., showed a significant but low correlation between PDvol and MM obstruction score (*r* = 0.33) but no correlation between PDvol and the axial RV/LV diameter ratio using a semiautomatic method to assess perfusion defects [20]. Apfaltrer et al., proposed that preserved perfusion in segmental arteries with partial occlusion might be a possible explanation to the low correlation between perfusion defect and obstruction score [20].

Several other articles have also reported a moderate to high correlation between perfusion defect score with both obstruction score and RV/LV diameter ratio while using a manual scoring method to assess perfusion defect score [11,12,21,22]. Perez-Johnston et al. reported a significant association between the presence of perfusion defect and several parameters of right heart strain (including RV/LV diameter ratio) and that higher perfusion defect score (using a manual scoring method) were related to higher mortality, when adjusted by cancer stage [23].

Automatically quantified PDvol were lower than semiautomatic quantified PDvol in most patients (*n* = 20) with acute PE (Figure 5). The automatic quantification seems to underestimate relative hypo-perfused areas that are not included as perfusion defects. Furthermore, the automatic quantification algorithm estimates suboptimal in the lower PDvol spectrum, as automatic PDvol seems to have a minimal threshold value of around 2% (lowest value recorded was 1.6%) regardless of acute PE findings or not.

It is worth noting that on a per-patient basis in the group with confirmed acute PE, some of the patients (*n* = 6) had low automatically quantified PDvol (≤2%), despite relative high MM obstruction scores (Figure 5). Therefore, patients with acute PE and patients without acute PE may both present a low automatic PDvol (≤2%) indicating that a low automatic PDvol cannot be used to solely reject the acute PE diagnosis. On the other hand some patients (*n* = 4) in the group without detectable acute PE (MM score = 0) on CTA had high automatically quantified PDvol of > 10% (Figure 5), but when assessed with semiautomatic PDvol, these patients had almost no wedge-shaped perfusion defects (close to 0%), suggesting that the defects seen on automatic PDvol were not associated with acute PE. Therefore, patients with high automatic PDvol cannot be used solely to diagnose acute PE. A possible explanation to this irregularity might be the scanner we used (GE Healthcare, Revolution CT with single source rapid kV switching technique), Singh et al. showed that this scanner had frequent artifactual heterogeneity when assessing pulmonary perfusion and therefore might impact the evaluation of atypical or subsegmental perfusion defects [24].

There were several limitations in our study. Interobserver variation in terms of reproducibility could not be measured as one radiologist assessed obstruction score and RV/LV diameter ratio while another radiologist assessed semiautomatic PDvol. No evaluation of experience level was assessed, and the results of this study may therefore only apply for readers with similar experience. We did not compare RV/LV diameter ratio or PDvol to their respective reference standard: echocardiography and lung perfusion scintigraphy. Data were not correlated with patient outcome, clinical or laboratory PE severity parameters. A recent study showed that DECT iodine maps had a strong correlation (*r* = 0.93) with single-photon emission computed tomography (SPECT-CT) in the quantification of relative lobar perfusion [25]. DECT pulmonary perfusion was not compared to SPECT-CT in our study. DECT created pulmonary perfusion maps were created based on iodine distribution at the specific time point of the scan, therefore a number of technical variables may have influenced the end result, e.g., contrast volume, iodine concentration, flow rate and scan timing. Hence, the relative perfusion deficit presented may not reflect a true biological perfusion deficit [11]. Our study was limited by the small population and a retrospective study design. Large sample size in a prospective designed study with machine learning software combined with clinical data would be desirable for future studies of DECT perfusion defect estimation of patients with acute PE.

## 5. Conclusions

In this small retrospective study, our data indicate that PDvol assessed by automatic and semiautomatic DECT software technique may be a helpful tool to assess the severity of acute PE when compared to obstruction score, while only semiautomatic PDvol may assess the severity of acute PE when compared to RV/LV diameter ratio.

## Data Availability

The data presented in this study are available on request.
